# Elastic Net Models Based on DNA Copy Number Variations Predicts Clinical Features, Expression Signatures, and Mutations in Lung Adenocarcinoma

**DOI:** 10.3389/fgene.2021.668040

**Published:** 2021-05-31

**Authors:** Yi Xiang, Xiaohuan Zou, Huaqiu Shi, Xueming Xu, Caixia Wu, Wenjuan Zhong, Jinfeng Wang, Wenting Zhou, Xiaoli Zeng, Miao He, Ying Wang, Li Huang, Xiangcai Wang

**Affiliations:** ^1^Department of Oncology, The First Affiliated Hospital, Gannan Medical University, Ganzhou, China; ^2^Department of Critical Care Medicine, The First Affiliated Hospital, Gannan Medical University, Ganzhou, China; ^3^First Clinical Medical College, Gannan Medical University, Ganzhou, China

**Keywords:** Elastic Net, DNA copy number, lung adenocarcinoma, gene expression signature, predictive model

## Abstract

In the precision medicine of lung adenocarcinoma, the identification and prediction of tumor phenotypes for specific biomolecular events are still not studied in depth. Various earlier researches sheds light on the close correlation between genetic expression signatures and DNA copy number variations (CNVs), for which analysis of CNVs provides valuable information about molecular and phenotypic changes in tumorigenesis. In this study, we propose a comprehensive analysis combining genome-wide association analysis and an Elastic Net Regression predictive model, focus on predicting the levels of many gene expression signatures in lung adenocarcinoma, based upon DNA copy number features alone. Additionally, we predicted many other key phenotypes, including clinical features (pathological stage), gene mutations, and protein expressions. These Elastic Net prediction methods can also be applied to other gene sets, thereby facilitating their use as biomarkers in monitoring therapy.

## Introduction

The enormous genetic heterogeneity caused by multiple types of DNA aberrations is a key factor in tumorigenesis. Thus, the capacity to analyze tumor inhomogeneity is essential to elucidating cancer etiopathogenesis and to more accurately defining patient subgroups in precision medicine (Gao et al., 2016; Godek et al., 2016; Padmanabhan et al., 2017; Pommier et al., 2020). One limitation that currently limits us to evaluate the heterogeneity is the accurate description of tumor phenotypes. The establishment of various data integration and analysis platforms, e.g., the Cancer Genome Atlas (TCGA), has enabled us to take advantage of readily accessible large-scale genomic data from multiple platforms to better analyze the association between gene expression heterogeneity and key phenotypes in the process of tumorigenesis (Hoadley et al., 2018). In particular, numerous reports have gradually uncovered various gene expression signatures that can be used for delineating specific tumor phenotypes from invasion rates to characterization of tumorous immune microenvironment (Bild et al., 2006; Nevins and Potti, 2007). These messenger RNA expression signatures, besides protein expressions, genetic mutation loads, and clinical characteristics, offer a wide-ranging cancer molecular representation (Kumar et al., 2018; Chen Y. J. et al., 2020). Therefore, the application of genomic data from these data analysis platforms to illuminate the connection between genotypes and phenotypes is significant to identify the genomic changes that occur during tumorigenesis (Gatza et al., 2014; Watermann et al., 2020; Zengin and Önal-Süzek, 2020). Furthermore, the predictions of phenotypes that drive tumorigenesis according to DNA expression status would be valuable to the stratification of patients in precision medicine (George et al., 2019; Carrillo-Reixach et al., 2020; Tsimberidou et al., 2020), specifically in current clinical practice, due to the expensive expression profile and the routine acquisition of genetic mutation information.

Lung cancer is a kind of malignancies with highest morbidity and mortality in the world (Siegel et al., 2019). About 85% of lung cancers are Non-small cell lung cancer (NSCLC), with an only approximately 16% 5-year survival rate (Namani et al., 2019). Lung adenocarcinoma (LUAD) is the most common histological subtype of NSCLC (Zhu et al., 2019). Extensive research in recent years has led to great advances in exploring the oncogenesis and treatment strategies of LUAD, however it remains one of the most deadly and metastatic types of lung cancer (Denisenko et al., 2018). Patients with LUAD often do not respond well to conventional radiotherapy or chemotherapy, because of the delay diagnosis in the middle and advanced stages. Therefore, it is still urgent to elucidate the pathogenic and molecular mechanism of LUAD. The ubiquitous DNA copy number variations (CNVs) in the human genome, containing amplifications, losses, insertions and multisite mutations, plays a critical role in the occurrence and developmentof various tumor types (López et al., 2020; Staaf et al., 2013). Specifically, CNV in tumor tissues and cells can lead to irregular expressions of tumor-related genetic drivers as well as genomic and molecular phenotypic heterogeneity (Kim et al., 2020). Previous studies have shown that DNA CNVs are associated with higher risks and poorer prognosis of LUAD. It is increasingly recognized that therapy strategies for LUAD should focus on the relationship between mutation loads and epigenetic alterations. However, there is still a lot of room for exploration. TP53, EGFR and KRAS mutations, for example, are critical in the occurrence and development of lung cancers, but not all tumors are caused by the activation of these mutations alone and eliminated by the inhibition of these genes (Jänne et al., 2015; Kobayashi et al., 2005). Here, we used gene expression signatures downloaded from the TCGA platform, together with common clinical and molecular features, as the basis for constructing predictive models of lung adenocarcinoma phenotype. We adopted an comprehensive genomic method, containing genome-wide association analysis (Gatza et al., 2014; Watermann et al., 2020), and an ElasticNet-mediated regression approach (Zou and Hastie, 2005), to modeling complicated tumorous phenotypes based on DNA copy number variations (CNVs). By these findings, we aim to elucidate the significant correlations of CNVs with several genetic expression signatures and protein expression landscapes. In general, our proposed method can be used to link CNVs with multifaceted phenotypes, also to develop evaluation models for therapeutic significance using currently commonplace DNA-based clinical tools.

## Materials and Methods

### Data Acquisition

Gene mutation data, protein expression data, and clinical information of lung adenocarcinoma samples in TCGA were downloaded from Xena^1^ (Hoadley et al., 2018). For TCGA lung adenocarcinoma, we converted the gene expression data using the upper quartile scale and log2 transform, and then screened for genes expressed in more than 70% of the samples.

### DNA CNVs Data

The collection, conversion and subsequent analysis of DNA copy number data were all based on GISTIC2.0 module. GISTIC2 gene-level CNVs information of human lung adenocarcinoma downloaded from TCGA GDAC FireBrowse (Mermel et al., 2011; Hoadley et al., 2018)^2^ are shown in Table 1, with no further processing, and the related clinicopathological characteristics are shown in Table 2. Using Ensembl 54 (hg18) genome build, gene-level copy number scores were derived through the extreme method as used in GISTIC2: Genes that fell completely within a circular binary segmentation (CBS)-identified copy number segment were assigned corresponding segment value. Genes that overlapped with multiple segments were assigned the greatest amplification or the least deletion value among the overlapped segments. Genes with no overlapping segments were excluded from further analyses (Xia et al., 2019).

**TABLE 1:** Sample information.

Data	Sample size
Mutation	513
CNV	516
Protein	237

**TABLE 2:** Patient and clinicopathological characteristics.

Characteristics	Types	Sample numbers
Gender		
	Female	275
	Male	237
Age (years)		
	>60	338
	≤60	155
Tumor invasion		
	T1	169
	T2	276
	T3	46
	T4	19
	Tx	2
Lymph node metastasis		
	N0	330
	N1	95
	N2	74
	N3	2
	Nx	10
	Not reported	1
Metastasis		
	M0	345
	M1	25
	Mx	138
	Not reported	4
TNM stage		
	Stage I	274
	Stage II	122
	Stage III	83
	Stage IV	25
	Not reported	8
Radiation_therapy		
	No	369
	Yes	57
	Not reported	86

### Gene Expression Signatures

Gene expression signature scores for 512 lung adenocarcinoma cases are composed of a panel of 531 previously published genetic expression signature, collected from a variety of earlier research, GSEA (Subramanian et al., 2005), and were partly summed up by Tanioka et al. (2018) that can be applied to completely describe tumor phenotypes. As for genetic signature scores, they were calculated in a manner consistent with their derivation. For 492 signatures with homogeneous expression across the genes, median expression value was used as signature score. The rest of the signatures were based on correlation to predetermined gene centroids or based on published algorithms. For correlation-based signatures, all predetermined training sets are available to download through our GitHub repository (see section Code Availability) (Xia et al., 2019). For each such signature, DWD was used to first merge gene expression matrix with corresponding training set and then Pearson/Spearman correlation/Euclidean distance was computed for each sample in the merged data. For several algorithm-based signatures, corresponding R code is provided to calculate each signature (see section Code Availability). All 531 signatures were applied to TCGA lung cancer data.

### Identification of Gene Signature-Specific CNVs

For identifying the association between CNVs and gene expression signatures, we employed two different statistical approach (Gatza et al., 2014; Watermann et al., 2020) on gene expression of TCGA lung adenocarcinoma cohorts and matched copy number data; the first involved calculating the Spearman correlation between the signature score and gene-level CNV score for each sample; the second statistical test involved performing a one-sided Fishers exact test for comparing the frequencies of CNV amplifications/deletions between higher score samples (top quartile) and lower score ones. The Benjamin-Hochberg (BH) method was applied to correct the *q*-value for each gene signatures for all analysis. The critical value of significance for the two statistical tests above (*q*-value) was set at 0.01.

### Construction of Elastic Net Regression Predictive Models

The Elastic-Net regression method, a linear combination of L1/L2 regularization in the Ridge regression and LASSO (Least absolute shrinkage and selection operator) regression, was utilized for building the CNV-based tumor phenotype predictions (Zou and Hastie, 2005). First, gene-level CNV scores were converted into segment-level CNV scores, which were averaged as the mean CNV score for all genes in this DNA segment (Beroukhim et al., 2010; Zack et al., 2013; Cancer Genome Atlas Research Network, 2014; Qiu et al., 2018; Hua et al., 2020). To construct the model, the total sample was split 70%:30% for the training and the test sets, respectively, which were also stratified by clinical variates (i.e., overall survival, sex, TNM stage and histopathological stage). Only the training set was used to build the models. The fitted generalized linear models were used to determine the maximum and minimum observed values of λ for each α value in the training sets (CRAN Rpackage glmnet) (Candia and Tsang, 2019). Using the default parameters, two hundred turns of MonteCarlo cross-validation were performed for screening the tuning parameter (Bandalos, 1993), and the optimal parameter was confirmed as the most accurate classification method. The models with the optimal parameters were subsequently used in the validation sets, and the area under the receiver operating characteristic curve (AUC) was applied to assess the predictive value of models. The phenotypes with AUC values higher than 0.75 were considered as with high predictive performance.

Since the variables involved in the prediction in this study are continuous data, we divided the sample into two-halves (using the top quartile and bottom three quartiles) to perform model predictions. For clinical characteristics and gene mutation status, which have binary outcomes, the model was built directly for their prediction. For gene mutation prediction, we selected the top 20 genes with the highest mutation frequencies in lung adenocarcinoma. Clinical features included pathological stage (stage I-II vs. stage III-IV) and TNM stage.

### Statistical Analysis

SPSS software (version 23.0) or R software (version 4.0.3) were used for statistical analysis. Curve analysis of receiver operating characteristic (ROC) was utilized to evaluate the predictive performance of CNV-based Elastic Net regression models. The AUC, ranging from 0.5 (for an uninformative marker) to 1 (for a perfect predictive marker), was a measurement for how well patient survival can be predicted with the gene signatures.

## Results

### Identification of DNA CNVs Associated With Gene Signatures

**FIGURE 1:**
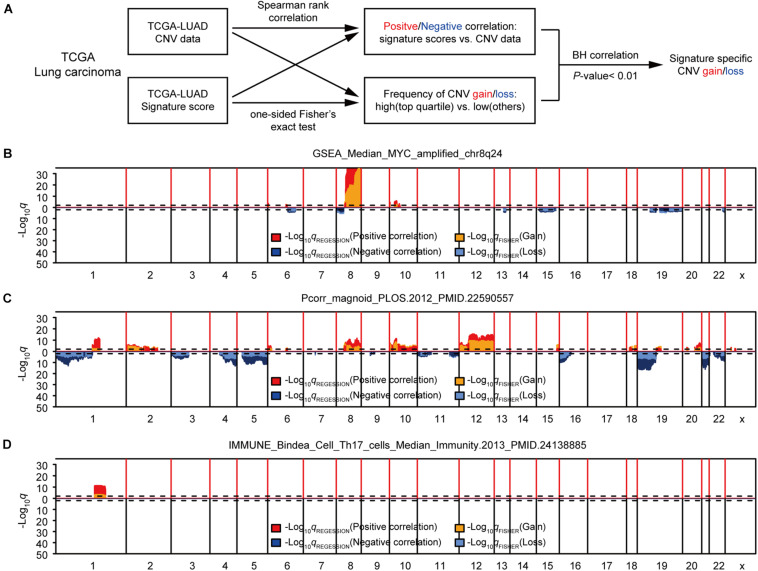
Identification of gene expression signature-specific CNVs in lung adenocarcinoma. **(A)** Schematic overview of the strategy used to identify CNVs associated with gene signatures. Gain/loss indicates DNA copy number gains or losses; Positive/Negative indicates positive or negative association. **(B,D)** Spearman rank correlation was used to identify genes that were positively (red) or negatively (dark blue) correlated with gene signatures, and Fisher’s exact test was used to compare the frequency of copy number gains (orange) or losses (light blue) in GSEA_Median_MYC_amplified_chr8q24 **(B)**, Pcorr_magnoid_PLOS.2012 **(C)**, and IMMUNE_Bindea_Cell_Th17_cells_Median_Immunity.2013 **(D)**. Dashed lines indicate the significance threshold (*q* = 0.01). Only the *q* values for genes that were significant in both analyses were plotted. In each figure, chromosome boundaries are indicated by vertical black lines.

First, we studied the potential correlations between multiple gene expression characteristics and gene CNVs. We used a group of 531 gene expression signatures reported previously to quantify various tumor phenotypes, including, but not limited to, tumor microenvironment characteristics and activated signaling pathways (Supplementary Table 1; Hoadley et al., 2018). We next applied a genome-wide association analysis to identify possible connections between each signature-based phenotype and DNA copy number data (Gatza et al., 2014; Watermann et al., 2020). Two different statistical methods were used for each signature to assess the correlation between DNA CNVs and the genetic signature for each genetic trait: The Spearman’s correlation was used for identifying negative/positive correlations between gene-level CNV and expression signature scores, and the Fisher’s exact test was performed to compare the differences in the probability of CNV gains/losses between the groups with higher expression signature scores (top 1/4) and low scores (other 3/4). B–H correction of *q* values were conducted for both sets of statistical methods (Benjamini and Hochberg, 1995). To further enhance accuracy, a given CNV was considered significantly associated with an expression signature only if the corrected *q* values for both statistical methods were < 0.01 (Figure 1A). Potential CNV drivers of a gene signature should increase the frequencies of CNV amplifications in samples with high signature scores and be positively correlated with its signature score. We used the methods above to analyze the relationship between each gene signature and CNV, and found that some gene signatures were significantly associated with CNVs, such as GSEA_Median_MYC_amplified_chr8q24, Pcorr_magnoid_PLOS.2012, and IMMUNE_Bindea_Cell _Th17_cells_Median_Immunity.2013 (Figures 1B–D and Supplementary Table 2). Taken together, this part of our study determined that the modeling methods could accurately find links between CNVs and specific genetic signatures, some had been confirmed in previous reports.

### Elastic Net-Mediated CNV-Based Predictions of Gene Signatures

**FIGURE 2:**
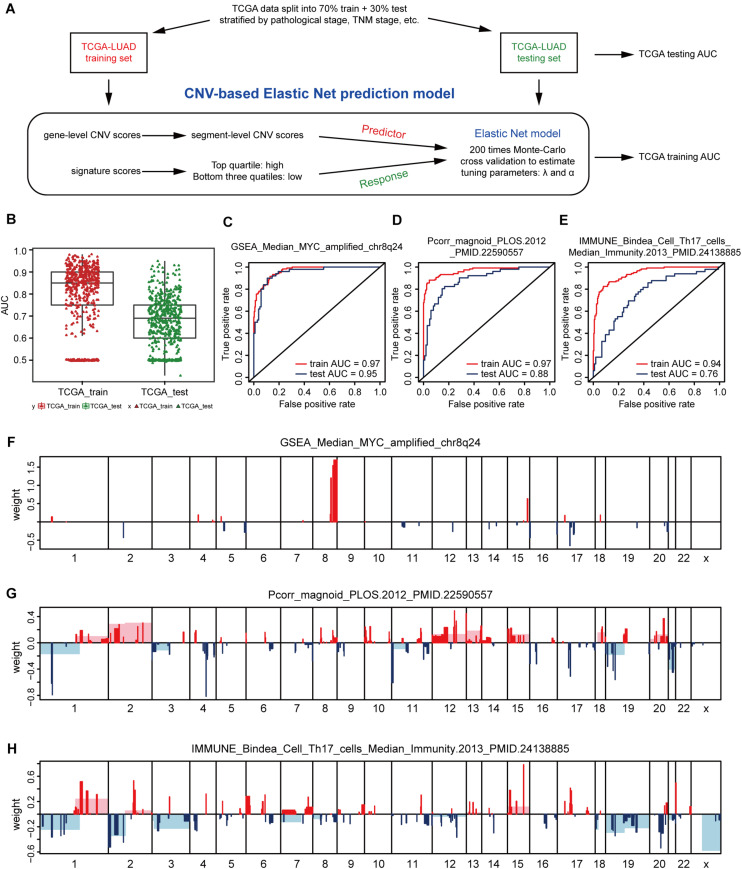
CNV-based gene signature prediction. **(A)** Schematic overview of the strategy used to build Elastic Net regression models for predicting gene expression signature levels. **(B)** AUC distributions of CNV-based prediction for the 531 gene signature scores of lung adenocarcinoma in the TCGA training and test set. **(C–E)** ROC curves and corresponding AUC values for three signatures in the training and test sets. GSEA_Median_MYC_amplified_chr8q24 **(C)**, Pcorr_magnoid_PLOS.2012 **(D)**, and IMMUNE_Bindea_Cell_Th17_cells_Median_Immunity.2013 **(E)**. **(F–H)** CNV segments and whole chromosomal arms and their corresponding coefficients selected by the Elastic Net prediction models for the three signatures.

In view of the strong relationship, we next constructed models that can predict the levels of genetic expression signatures on the basis of DNA CNVs landscapes alone. For constructing the predictive models, a modeling method known as Elastic-Net was utilized, which can handle several potential co-linear variables that are present in regression models, and then screen out the most related elements for the final regression modeling (Zou and Hastie, 2005). Gene-level CNV scores were not used during model building, instead we applied the segment-level CNV scores of chromosomal regions proven being significant in multiple tumor types (Beroukhim et al., 2010; Zack et al., 2013; Qiu et al., 2018; Hua et al., 2020; Supplementary Table 3). The 512 cases of lung adenocarcinoma were divided 70% into the training group and 30% into the validation group. Models were constructed on the TCGA training set, and tested on the validation set, and model performance was assessed using AUC value (Figure 2A).

The AUC value of some genetic signatures indicated that the CNV-based Elastic Net prediction models can be good forecasters of certain gene signatures (Table 3 and Supplementary Table 4_AUC_signature). Among the 531 gene signatures, 142 in the test set had AUCs > 0.75 (Figure 2B), henceforth denoted as “highly predictable.” Of these 142 signatures, only 21 were DNA-based amplicon signatures that essentially measure specific CNA events and were therefore expected to produce high AUC values. For example, signature 11q13-amplicon had a fairly high AUC value in test set (AUC = 0.90). Notably, the three signatures highlighted in the previous correlation analysis, namely, GSEA_Median_MYC_amplified_chr8q24, Pcorr_magnoid_PLOS.2012, and IMMUNE_Bindea_Cell_Th17_cells_Median_Immunity.2013 were all highly predictable and their corresponding AUC were 0.95, 0.88, and 0.76, respectively (Figures 2C–E). The results of this part show the prediction efficiency of the Elastic Net model.

**TABLE 3:** Top 20 AUC value of gene signatures predicted by Elastic Net models.

Rank	Gene signatures	Train set	Test set
1	GSEA_Median_MYC_amplified_chr8q24	0.97	0.95
2	GP19_1Q_amplicon.PerouLab_HS_Green17 Median_BMC_Med_Genomics	0.95	0.93
3	UNC_HS_Green17_Median_BMC.Med.Genomics.2011_PMID.21214954	0.95	0.93
4	IntClust_Deletion.50.Better.than_Median_Genome.Biol.2014_PMID.25164602	0.94	0.93
5	UNC_HS_Red15_Median_BMC.Med.Genomics.2011_PMID.21214954	0.93	0.93
6	UNC_8p_Amplicon_Median_BMC.Med.Genomics.2011_PMID.21214954	0.95	0.92
7	UNC_HS_Green24_Median_BMC.Med.Genomics.2011_PMID.2121495	0.93	0.92
8	UNC_8p22_Amplicon_Median_BMC.Med.Genomics.2011_PMID.21214954	0.97	0.91
9	UNC_HS_Red14_Median_BMC.Med.Genomics.2011_PMID.21214954	0.93	0.91
10	UNC_11q13_Amplicon_Median_BMC.Med.Genomics.2011_PMID.21214954	0.93	0.91
11	UNC_17PP13_Amplicon_Median_BMC.Med.Genomics.2011_PMID.21214954	0.95	0.90
12	Scorr_PTEN_Absent_Correlation_PNAS.2007_PMID.17452630	0.93	0.90
13	UNC_Scorr_P53_Wt_Correlation_BMC.Cancer.2006_PMID.17150101	0.90	0.90
14	UNC_15q25_Amplicon_Median_BMC.Med.Genomics.2011_PMID.21214954	0.97	0.89
15	UNC_17q25x_Median_BMC.Med.Genomics.2011_PMID.21214954	0.96	0.89
16	Pcorr_magnoid_PLOS.2012_PMID.22590557	0.93	0.89
17	UNC_4p16_Amplicon_Median_BMC.Med.Genomics.2011_PMID.21214954	0.93	0.89
18	UNC_Scorr_IE_Correlation_JCO.2006_PMID.16505416	0.97	0.88
19	IntClust_Deletion.50_Median_Genome.Biol.2014_PMID.25164602	0.95	0.87
20	UNC_13q14_Amplicon_Median_BMC.Med.Genomics.2011_PMID.21214954	0.93	0.86

For comparing these associated landscapes, the CNV regions and entire chromosome arms and the coefficients selected by Elastic Net models of the three features above are shown in Figures 2F–H (Supplementary Table 4). Remarkably, Pcorr_magnoid_PLOS.2012, and IMMUNE_Bindea_Cell_Th17_cells_Median_Immunity.2013 signatures had a large amount of correlation landscape with CNVs (Figures 2G,H). On the contrary, the correlation characteristics of GSEA_Median_MYC_amplified_chr8q24 signature had little intersection with those of the Elastic Net model (Figure 2F). In general, these consequences indicate that DNA CNV features can be used to predict gene expression signatures.

### Elastic Net-Mediated CNV-Based Predictions of Protein Expressions

**FIGURE 3:**
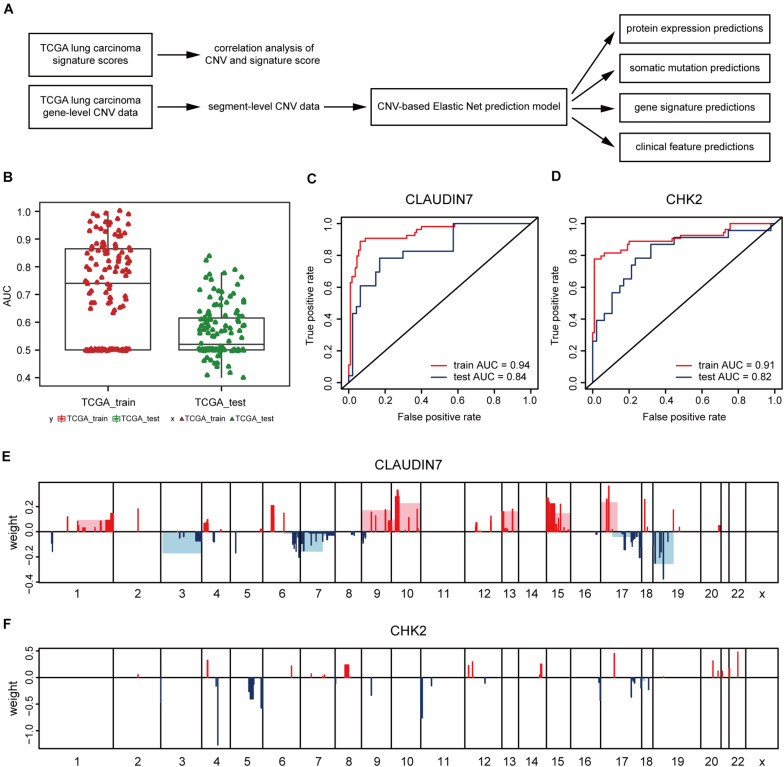
CNV-based protein expression prediction. **(A)** Flowchart showed the construction of CNV-based Elastic Net prediction models and the certification of its predictive value on gene expression, protein, mutation and clinical features. **(B)** AUC distributions of CNV-based expression level predictions for 131 proteins in the RPPA arrays. **(C,D)** ROC curves and corresponding AUC values of Claudin7 **(C)** and CHK2 **(D)** in the TCGA training and test sets. **(E,F)** Elastic Net-selected CNV segments and whole chromosomal arms and their coefficients for prediction models for the two proteins.

Next, the method of Elastic Net-mediated CNV-based prediction was used for constructing models of specific protein expressions. Reverse phase protein array (RPPA) information was used (Network, Cancer Genome Atlas Research, 2012; Cancer Genome Atlas Research Network, 2014) to measure the protein expression levels of TCGA lung adenocarcinoma samples (Figure 3A). A few studies have reported that DNA copy number has a significant impact on the expressions of some proteins (Geiger et al., 2010; Myhre et al., 2013; Liu et al., 2016). However, these studies only assessed the correlations with proteins encoded by individual genes. Here, Elastic Net predictive modeling was applied to account for genome-wide CNV changes in the prediction of protein expressions. By setting the AUC at > 0.75 as “high predictive value,” (Figure 3B) 8 of 131 proteins expression levels can be predicted with high accuracy (Table 4 and Supplementary Table 5), containing Claudin7 and CHK2 (Figures 3C,D). Notably, Beroukhim features 17p and 15q deletions were involved within the models, in agreement with the condition that Claudin7 negativity is common among patients with lung cancers (Figure 3E; Lu et al., 2015; Choi et al., 2016; Akizuki et al., 2017). Similarly, the result in Figure 3F was consistent with other previous reports about the lower expression of CHK2 in adenocarcinoma (Chen et al., 2012; Choi et al., 2012). Thus, our method can accurately predict these complex protein expressions based on a little fraction of genomic information.

**TABLE 4:** Top 20 AUC value of protein expressions predicted by Elastic Net models.

Rank	Proteins	Train set	Test set
1	CLAUDIN7	0.94	0.84
2	CHK2	0.91	0.82
3	CKIT	0.98	0.79
4	CYCLINB1	0.86	0.78
5	ASNS	0.96	0.77
6	ERALPHA	0.99	0.76
7	PKCALPHA	0.74	0.76
8	4EBP1	0.80	0.75
9	MTORPS2448	0.92	0.74
10	IGFBP2	0.95	0.72
11	EGFRPY1173	0.92	0.72
12	INPP4B	0.87	0.71
13	MRE11	0.75	0.71
14	JNK2	0.85	0.70
15	PDK1PS241	0.93	0.69
16	GSK3ALPHABETA	0.85	0.69
17	JNKPT183Y185	0.83	0.69
18	P70S6K	0.72	0.68
19	RBPS807S811	0.86	0.67
20	ACCPSCPS79	0.86	0.67

### Elastic Net-Mediated CNV-Based Predictions of Mutation Loads

**FIGURE 4:**
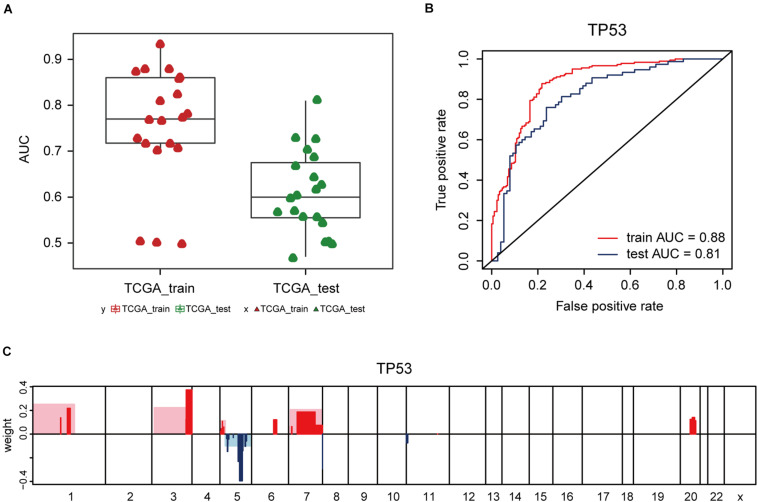
CNV-based genetic mutation prediction. **(A)** AUC distributions of CNV-based predictions for top 20 genes with the highest mutation frequencies selected from TCGA lung adenocarcinoma mutation data. **(B)** ROC curves and corresponding AUC values of TP53 mutation status in the TCGA training and test sets. **(C)** Elastic Net-selected CNV segments and whole chromosomal arms and their coefficients selected for prediction models for TP53 mutation.

Additionally, the capacity of CNV-based Elastic Net predictive models to foretell somatic mutation was tested. We first downloaded the mutation information of lung adenocarcinoma samples in TCGA database (Hofree et al., 2013), from which the top 20 genes with the highest mutation frequencies were selected (Figure 4A and Table 5). By setting the AUC threshold of validation set as 0.75, only the TP53 mutation was screened out (Figure 4B), corresponded with its widely recognized role as a cancer suppressor. And the differential expression of PIK3CA induced by Beroukhim 3q gain was speculated to take part in the tumorigenesis of lung adenocarcinoma when TP53 silencing (Yang et al., 2014; Hou et al., 2020; Wang F. et al., 2020; Figure 4C and Supplementary Table 6_TP53_weight). Moreover, when the AUC threshold was relaxed to 0.7, the TP53, RYR2, TTN, LRP1B and CSMD3 mutations satisfied the condition (Supplementary Table 6_AUC_snp), indicating their correlations with increased tumor mutation burden.

**TABLE 5:** Top 20 AUC value of somatic mutations predicted by Elastic Net models.

Rank	Proteins	Train set	Test set
1	TP53.txt	0.88	0.81
2	RYR2.txt	0.77	0.73
3	TTN.txt	0.77	0.73
4	LRP1B.txt	0.87	0.70
5	CSMD3.txt	0.86	0.69
6	KRAS.txt	0.93	0.67
7	SPTA1.txt	0.72	0.64
8	USH2A.txt	0.77	0.63
9	PCDH15.txt	0.81	0.62
10	MUC16.txt	0.86	0.60
11	NAV3.txt	0.78	0.60
12	CSMD1.txt	0.70	0.57
13	XIRP2.txt	0.88	0.57
14	ANK2.txt	0.72	0.56
15	FLG.txt	0.73	0.56
16	ZNF536.txt	0.82	0.54
17	COL11A1.txt	0.50	0.50
18	FAT3.txt	0.50	0.50
19	ZFHX4.txt	0.50	0.50
20	MUC17.txt	0.71	0.47

### Elastic Net-Mediated CNV-Based Predictions of Clinical Features

**FIGURE 5:**
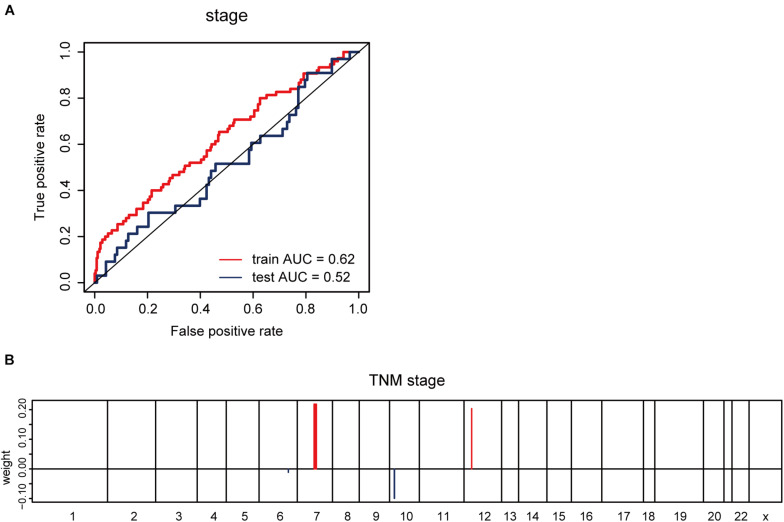
CNV-based prediction of clinical features. **(A)** ROC curves and corresponding AUC values for CNV-based prediction of clinical stage (stage I–II vs. stage III–IV) in the TCGA training and test sets. **(B)** Elastic Net-selected CNV segments and whole chromosomal arms and their coefficients for prediction model for pathological stage.

Furthermore, we explored whether CNV-based Elastic Net predictive regression models can be used to forecast clinical features. Here, pathological stage and TNM stage were selected to divide the sample into two halves (stage I –II, as one group; stage III –IV, as another). After grouping the samples for model building and prediction, the AUC of the prediction results was found to be 0.52 (Figure 5A). The model feature landscapes for TNM stage were shown in Figure 5B. For model building and prediction based on TNM stage, all AUCs were 0.5. Additionally, we acquired radiotherapy_information for TCGA lung adenocarcinoma samples, and applied the Elastic Net predictive model, but the prediction outcomes were poor, and all AUCs were 0.5.

## Discussion

In this study, we applied chromosomal segment-level CNVs downloaded from TCGA database to perform Elastic Net predictive model building, and the resulting models were used for the forecasting of mutations, protein expression, clinical features, and gene signature scores in lung adenocarcinoma.

The ability to predict key tumor phenotypes is essential for elucidating the biocomplexity in multiple cancer types (Bailey et al., 2018; Ding et al., 2018). The standard treatment procedure for lung adenocarcinoma includes DNA-based gene expression profiling, whiles protein expression analysis (e.g., PDL1) is gaining prominence, which is mostly owing to immune therapy (Wang L. et al., 2020). Numerous previous studies have confirmed that lung cancer is likely to be driven by gene copy numbers in some degree due to the occurrence of numerous copy number events, many of which are known genetic drivers (Wilkerson et al., 2012; Campbell et al., 2016; Chen J. et al., 2020; Gillette et al., 2020). Therefore, we reasoned that when testing a copy number-driven tumor type, the diversity of DNA CNVs can be used to predict many key tumor phenotypes. To verify the above hypothesis, a wide-ranging manual archiving of arranged genetic expression signatures extracted from plenty of existing studies were utilized for analyzing tumorous phenotype and assess the predictability. Two methods were adopted to investigate the associations between every gene expression signature and DNA CNV; a genome-wide association analysis and Elastic Net regression modeling method. The correlation research was able to identify whether genes have positive or negative correlations with gene characteristics by evaluating the genes individually. The combination of the two methods synergistically produced a complete landscape of the connection between gene signature and CNV, including many known linkages, such as genetic signature for DNA gains and deletions, or some signaling pathway activities (e.g., TP53). Indeed, many of these signatures in the true validation group were highly predictable (AUC > 0.8). Combining the association landscape for each gene marker with its Elastic Net features has provided us with exact CNV region for deeper exploration of possible genetic drivers for multiple tumors. Besides, the following usage of Elastic Net predictive model method in many other phenotypes, such as the levels of proteins and mutations, has demonstrated the capacity to forecast various essential phenotypes of lung adenocarcinoma with high accuracy.

Nowadays, many similar studies are based on the expression levels of related genes to build the prediction models for molecular expression signatures, clinical indicators, disease progression and prognosis. However, our study starts from the perspective of DNA copy number data, intending to excavate the potential information contained in which, and predicting key tumor phenotypes, like mutation status or biomarker levels or complex expression phenotypes in a proposed copy number-driven tumor type, e.g., lung adenocarcinoma. In addition, instead of some simple methods such as Logistics regression and random forest used in some other conventional prediction model researches, the DNA CNVs-based prediction models in our study are built on the basis of Elastic Net regression. With the characteristics of both Ridge regression and LASSO regression, the Elastic Net algorithm can effectively achieve the screening of eigenvectors with group effect and compress the selected variables to avoid the model over-fitting, so as to maintain the simplicity and accuracy of the model at the same time.

This modeling strategy might have clinical benefit and offer an orthogonal method to addressing significant features like TP53 status, especially considering the increasing value of gene exons and genetic mutations goes with the diagnosis and treatment of tumors (Kinde et al., 2013; Garofalo et al., 2016; Uzilov et al., 2016; Valle et al., 2020). Our study indicates that a little fraction of genomic information plays a significant role in predicting many molecular phenotypes in lung cancer. This also provides possibility for the clinical use of Elastic Net model. For instance, a variety of cell cycle progression and apoptosis signatures, such as c-MYC signature assessed above, may play a predictive role in the efficacy of NEDD8 inhibitors targeting PI3K/c-MYC axis (Ochiiwa et al., 2021), or for MAPK inhibitors by targeting ABL1/2-mediated reactivation of MEK/ERK/c-MYC signaling (Tripathi et al., 2020). The Elastic Net prediction models of c-MYC signature were able to extract out the patients with high proliferation rates, which generally accompanied by MYC amplification, then the use of NEDD8 inhibitors is recommended for these patients. If followed by more extensive trial and verification, it might be possible to read out plenty of novel prediction markers for cancer diagnosis, efficacy, and prognosis from available genomic panel information, hence playing a better guiding role in tumor personalized medicine without increasing the expense.

The strengths of this present study are that the model has been validated in both the test and validation sets, and that some results have previously been shown to be associated with lung adenocarcinoma, such as GSEA_Median_MYC_amplified_chr8q24 and Pcorr_magnoid_PLOS.2012. Nevertheless, there are still many shortcomings in our study. For example, in the prediction of clinical features, we analyzed the pathological stage, TNM stage, and radiotherapy, but only the prediction outcome of the pathological stage in the validation set was > 0.5. No new sample sets with both CNV and expression profile data were found for further validation, so we used 70% TCGA data for testing, and another part of the data was used for validation.

## Conclusion

Overall, our study exhibited the capability to construct CNV-based Elastic Net predictors for multiple key tumor phenotypes in patients with lung adenocarcinoma. Although most studies have focused on the search for genetic drivers of tumorigenesis, our results had the important connotation that DNA information can be used to predict significant complex tumor phenotypes, which could have applications in the clinical settings.

## Code Availability

All code supporting the current study is deposited in GitHub (https://github.com/xyouli/DNA-based-predictors-of-non-genetic-cancer-phenotypes). All computational analyses are done using public R packages.

## Data Availability Statement

The original contributions presented in the study are included in the article/Supplementary Material, further inquiries can be directed to the corresponding authors.

## Ethics Statement

The collection of human tissue samples or clinical data were not involved in this study. All data was downloaded from network open databases.

## Author Contributions

YX designed the study, wrote the manuscript, and took responsibility for the integrity of the data and the accuracy of the data analysis. YX and CW performed the database analysis from Xena and GSEA. YX, WZhon, and JW carried out the CNV identification. YX, HS, and XX did the CNV-based signature prediction. CW, WZhon, JW, WZhou, and XZe revised the manuscript. LH and XW planned and supervised all these works. All authors have read and approved the final manuscript.

## Conflict of Interest

The authors declare that the research was conducted in the absence of any commercial or financial relationships that could be construed as a potential conflict of interest.

## Footnotes

1 https://xenabrowser.net/datapages/

2 https://gdac.broadinstitute.org/#

## Abbreviations

CNVs: copy number variations
CNV: copy number variation
LASSO: Least absolute shrinkage and selection operator
TCGA: the cancer genome atlas
DFS: disease-free survival
OS: overall survival
RPPA: reverse-phase protein arrays
HR: hazard ratios
CI: confidence interval
ROC: receiver operating characteristic
AUC: area under the curve.
